# Activation of NRG1-ERBB4 signaling potentiates mesenchymal stem cell-mediated myocardial repairs following myocardial infarction

**DOI:** 10.1038/cddis.2015.91

**Published:** 2015-05-21

**Authors:** X Liang, Y Ding, Y Zhang, Y-H Chai, J He, S-M Chiu, F Gao, H-F Tse, Q Lian

**Affiliations:** 1 Department of Medicine, the University of Hong Kong, Hong Kong, China; 2 Organ Transplantation Institute, Xiamen University, Fujian Province, China; 3 Research Centre of Heart, Brain, Hormone, and Healthy Aging, Li Ka Shing Faculty of Medicine, the University of Hong Kong, Hong Kong, China; 4 Shenzhen Institutes of Research and Innovation, the University of Hong Kong, Hong Kong, China; 5 Hong Kong-Guangdong Joint Laboratory on Stem Cell and Regenerative Medicine, Hong Kong, China; 6 Department of Ophthalmology, Li Ka Shing Faculty of Medicine, the University of Hong Kong, Hong Kong, China

## Abstract

Mesenchymal stem cell (MSC) transplantation has achieved only modest success in the treatment of ischemic heart disease owing to poor cell viability in the diseased microenvironment. Activation of the NRG1 (neuregulin1)-ERBB4 (v-erb-b2 avian erythroblastic leukemia viral oncogene homolog 4) signaling pathway has been shown to stimulate mature cardiomyocyte cell cycle re-entry and cell division. In this connection, we aimed to determine whether overexpression of ERBB4 in MSCs can enhance their cardio-protective effects following myocardial infarction. NRG1, MSCs or MSC-ERBB4 (MSC with ERBB4 overexpression), were transplanted into mice following myocardial infarction. Superior to that of MSCs and solely NRG1, MSC-ERBB4 transplantation significantly preserved heart functions accompanied with reduced infarct size, enhanced cardiomyocyte division and less apoptosis during early phase of infarction. The transduction of ERBB4 into MSCs indeed increased cell mobility and apoptotic resistance under hypoxic and glucose-deprived conditions via a PI3K/Akt signaling pathway in the presence of NRG1. Unexpectedly, introduction of ERBB4 into MSC in turn potentiates NRG1 synthesis and secretion, thus forming a novel NRG1-ERBB4-NRG1 autocrine loop. Conditioned medium of MSC-ERBB4 containing elevated NRG1, promoted cardiomyocyte growth and division, whereas neutralization of NRG1 blunted this proliferation. These findings collectively suggest that ERBB4 overexpression potentiates MSC survival in the infarcted heart, enhances NRG1 generation to restore declining NRG1 in the infarcted region and stimulates cardiomyocyte division. ERBB4 has an important role in MSC-mediated myocardial repairs.

Although mesenchymal stem cell (MSC)-based cell transplantation is a promising and novel approach for cardiac repair following myocardial infarction (MI) that involves paracrine factors and cardiovascular differentiation,^1, 2, 3, 4^ the poor survival and engraftment of transplanted stem cells within the ischemic myocardium remain major limitations to this process. Numerous strategies have been used to improve MSC-based therapeutic potential, among which genetic modification has drawn considerable attention. Introducing genes into MSCs to enhance cell viability, mobility and angiogenesis has been explored.^5, 6, 7, 8^ For example, overexpression of the anti-apoptotic factor Bcl-2 (B-cell lymphoma 2) in MSCs enhances survival capacity and improves cardiac performance during MI following transplantation.^9^ Similarly, MSCs with IGF1 (insulin-like growth factor 1) overexpression induce stem cell mobilization and increase angiomyogenesis that subsequently contribute to myocardial repair.^10^ Nevertheless the effects of these genetically engineered MSCs on adult mature cardiomyocyte regeneration are unclear. Identification of novel genes or pathways that both ameliorate MSC properties and initiate endogenous cardiomyocyte regeneration *in situ* will thus provide crucial benefits.

Following examination of adult hearts, Bersell *et al.*^11^ recently revealed that NRG1 (neuregulin1) can stimulate mononuclear mature cardiomyocytes to re-enter the cell cycle and DNA duplicate status through the NRG1-ERBB signaling pathway. Strategic targeting of the signaling complex of NRG1 and its tyrosine kinase receptors, ERBB2 (v-erb-b2 avian erythroblastic leukemia viral oncogene homolog 2) and ERBB4 (v-erb-b2 avian erythroblastic leukemia viral oncogene homolog 4) has demonstrated positive outcomes following heart failure in animal models.^12, 13, 14^ NRG1, a member of the epidermal growth factor family, has been comprehensively documented as an essential paracrine regulator of cell–cell communication through activation of its ERBB tyrosine kinase receptors, and is indispensable in heart development and adult cardiac physiology.^11, 15, 16, 17, 18, 19^ The recombinant human NRG1 has been used in clinical trials to treat chronic heart failure in China and Australia (ClinicalTrials.gov identifiers NCT01131637, NCT01258387), in which it proved to enhance heart function and reverse remodeling.^20, 21^ ERBB2 acts as a non-ligand binding, pre-activated co-receptor that needs to heterodimerize with ERBB4 and forms an ERBB2/ERBB4 complex upon activation by NRG1. In contrast, ERBB4 possesses the capability of homodimerization and activates PI3K/Akt (v-akt murine thymoma viral oncogene homolog 1) signaling that facilitates cell survival. Although this crucial discovery suggests the potential of using NRG1 as a therapeutic agent for cardiac repair, numerous challenges must be addressed before it can become a reality. First, the circulating half-life of NRG1 is extremely short (~30 min),^14^ thus the interval during which it can be delivered is limited. Attempts have been made to prolong the NRG1 half-life by using a controlled delivery^22^ or by combining it with adipose-derived stem cell transplantation.^23^ Second, a relatively small population (10%) of cardiomyocytes in the adult heart are mononucleated and although systematic administration of NRG1 initiates cytokinesis *in vivo*, it only corresponds to ~0.3% of the mononuclear cardiomyocytes present following MI.^11^ Third, the receptor presenting on the targeting cells has a vital role in managing whether the cells respond to NRG1 stimuli or not. These sub-optimal results may attribute to the low penetration of NRG1 or in the ischemic myocardium that consequently limits its therapeutic efficiency.

Both MSC therapy and targeting the NRG1-ERBB complex hold great potential for treating cardiac diseases. Our hypothesis is therefore to combine these advantages by engineering MSCs and studying whether and how these cells can confer cardiac protection following transplantation into infarcted hearts. To this end, we first screened expression of ERBB receptors in mouse MSCs and determined that NRG1 and ERBB2, but not ERBB4, were expressed in MSCs. As ERBB2 is incompetent to bind NRG1, transplanting unmodified MSCs that lack ERBB4 cannot activate NRG1-ERBB signaling, and thus stultifies the existing NRG1 in the infarcted myocardium.^24^ We introduced ERBB4 expression into MSCs (MSC-ERBB4) and transplanted them into MI model. MSC-ERBB4 not only significantly preserved heart functions accompanied with reduced infarct size and enhanced cardiomyocyte division, but also improved MSC survival and created an autocrine loop in the NRG1-ERBB4-NRG1 signaling pathway. These results suggest that engineering ERBB4 in MSCs could be an novel strategy to benefit both MSCs and cardiomyocytes for enhancing efficacy of MSC-based transplantation in heart infarction.

## Results

### Expression of ERBB4 in MSCs didn't alter MSCs differentiation potential

**Figure 1:**
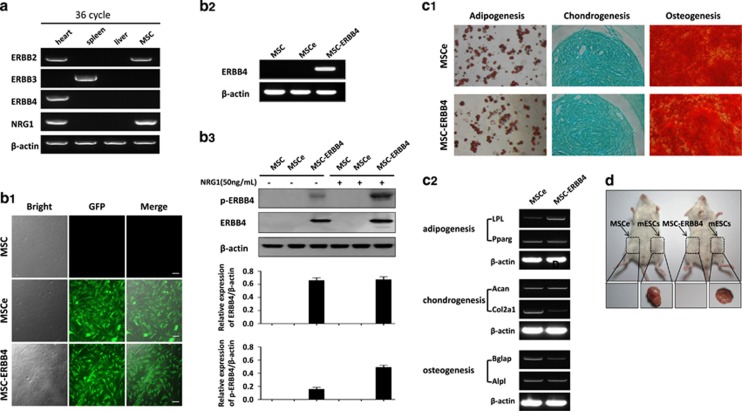
Exogenous ERBB4 was successfully transduced in MSCs. (**a**) RT-RCR screening of NRG1-ERBB expression in MSCs. MSC expressed NRG1 and ERBB2, but not ERBB4. (**b**) MSCs were successfully transduced with pGFP or pER4-GFP (map shown in Supplementary Figure 2), confirmed by using a GFP-positive signal detected under a fluorescent microscope (**b1**). RT-PCR (**b2**) and western blotting (**b3**) confirmed successful manipulation of ERBB4 into MSCs. Elevated p-ERBB4 occurred when additional NRG1 was added. p-ERBB4, phosphorylated ERBB4. (**c**) Lentiviral-transduced MSCe and MSC-ERBB4 possessed multi-lineage differentiation capacity, confirmed by staining (**c1**) and lineage specific gene expression (**c2**). (**d**) Neither MSCe nor MSC-ERBB4 injection raised malignant formation during 8 weeks of observation, with mouse embryonic stem cells as positive control. mESCs, mouse embryonic stem cells. *n*=4 for each group

MSCs isolated from tibias and spindle-like MSCs (Supplementary Figure 1) were *ex vivo* culturing until passage 4 to exclude non-adherent hematopoietic cells. The expression profile of ERBB family and its ligand NRG1 were examined by RT-PCR (reverse transcription polymerase chain reaction) following MSCs characterization (Supplementary Figure 1). MSCs were positive for NRG1 and ERBB2, but negative for ERBB3 and ERBB4 (Figure 1a). MSCs were transduced with a lentiviral vector containing the ERBB4 cDNA and GFP (green fluorescent protein; MSC-ERBB4) or GFP (MSCe; plasmid map showed in Supplementary Figure 2). Fluorescence signal was observed in both MSCe and MSC-ERBB4 (Figure 1b1). ERBB4 was detected in MSC-ERBB4, but not MSCe (Figure 1b2 and b3). The expression of phosphorylated ERBB4 (p-ERBB4), was increased in MSC-ERBB4 under NRG1 treatment (Figure 1b3). The multiple differentiation potential of adipogensis, chondrogenesis and osteogenesis was not affected after lentiviral manipulation (Figure 1c1 and c2). To determine the safety of introducing ERBB4 into MSCs, we tested the risk of malignant transformation. MSCe and MSC-ERBB4 were subcutaneously injected into NOD-SCID mice, with mouse embryonic stem cells (mESCs) serving as a positive control. After an 8-week observation period, neither MSCe nor MSC-ERBB4 induced tumorgenesis, whereas macroscopic tumor formation was observed at the mESCs injection site (Figure 1d).

### MSC-ERBB4 transplantation reduces infarction size and preserves heart function

**Figure 2:**
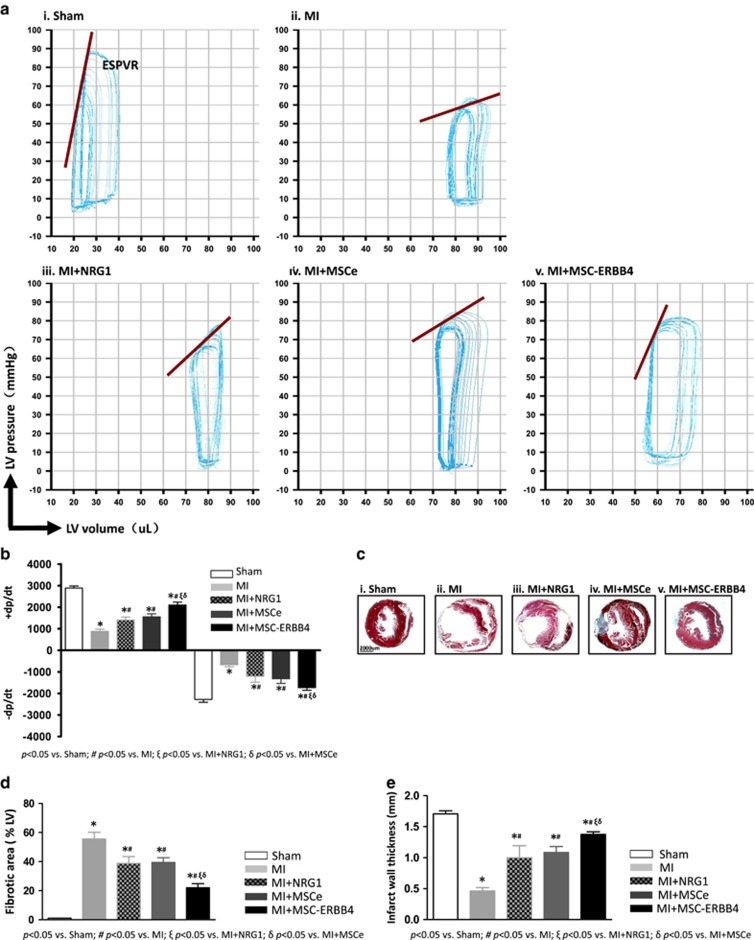
Transplantation of MSC-ERBB4 reduced fibrosis and improved heart function. Heart function was measured using a pressure-volume conductance catheter system 4 weeks after injection of NRG1, MSCe or MSC-ERBB4. (**a)** Representative single PV-loop recording images are shown. MI resulted in a rightward shift in the loop, which increased the volume and depression of the ESPVR slope (**aii** *versus* **ai**, red line). MSC-ERBB4 but not MSCe (**av** *versus* **aiv**) or NRG1(**av** *versus* **aiii**) reduced the volume and restored the original slope of the ESPVR. (**b)** Enhanced peak velocities of pressure change (dp/dt) occurred during isovolumic contraction (+dp/dt) and isovolumic relaxation (−dp/dt) in mice injected with MSC-ERBB4. (**c)** ERBB4 overexpression reduced scar formation following MI. Masson's trichrome staining indicated the thinning and expansion of the infarct scar (blue color) in the MI group (**cii** *versus* **ci**) and was attenuated in the MSC and NRG1 treated groups at 4 weeks post-MI (**ciii**, **civ** and **cv**). Representative photomicrographs for each group are shown. (**d**) Quantification of fibrotic area presented as the percentage of LV area positively stained with Masson trichrome. (**e**) Quantification of LV wall thickness. MSC-ERBB4 transplantation resulted in smaller infarct zone within the total LV area compared with that in the MI, MI+NRG1 and MI+MSCe groups and increased infarct wall thickness. LV, left ventricular; ESPVR, end-systolic pressure-volume relationship. *n*=12 for each group

To evaluate whether overexpressing ERBB4 generates an enhanced therapeutic effect of MSCs, we transplanted MSC-ERBB4, MSCe and saline controls into a mouse model of heart infarction. To demonstrate the advantages of MSC-ERBB4 therapy compared with the direct application of NRG1, another group of mice were treated with NRG1 injection following MI. A PV-loop (pressure-volume loop) provides continual assessment of LV (left ventricular) pressure and relative volume to indicate cardiac performance. An ESPVR (end-systolic pressure-volume relationship) refers to the maximal pressure that can be produced by the ventricle at any given LV volume, and provides an index of myocardial contractility. LAD (left anterior descending artery) ligation caused a shift to the right in the loop to increase the volume and depression slopes of the ESPVR (Figure 2aii versus ai, red line), indicating dilated cardiomyopathy, in accordance with the morphological changes shown in Figure 2cii. MSC-ERBB4 treatment reduced the volume and restored the slope of the ESPVR more than NRG1 or MSCe injection, indicating that a more powerful contractility occurred (Figure 2av versus aiii and aiv). The peak velocity of pressure change (dp/dt) is a reliable index used to measure ventricular function, because parameters such as afterload, wall motion abnormalities and variations in ventricular anatomy and morphology that appear in a diseased model do not affect the results. LV hemodynamic and volume data at week 4 is shown in Table 1. The data implied that the index for contractility that occurred during isovolumic contraction, +dp/dt, in the NRG1 and cell transplantation groups was much higher than that of the MI group (Table 1 and Figure 2b), whereas MSC-ERBB4 demonstrated superior performance to NRG1 or MSCe (Table 1 and Figure 2b, *P*<0.05 *versus* MI+NRG1, *P*<0.05 *versus* MI+MSCe). A similar pattern was observed in –dp/dt, an index for LV diastolic function (Table 1 and Figure 2b, *P*<0.05 *versus* MI+NRG1, *P*<0.05 *versus* MI+MSCe). These results showed that MSCs with ERBB4 expression reduced post-MI deterioration in cardiac function, with a cardio-protective effect significantly greater than that of solely NRG1 or MSCs without ERBB4.

Gross histological examination was performed using Masson trichrome staining, in which a fibrotic area was indicated by a blue color. The infarct size was quantified by the average ratio of fibrosis area to the total LV area (percent fibrosis area). The percentage fibrotic area in the MI group was transmural, occupying 55.4±4.5% of the LV (Figure 2cii and d), whereas the fibrosis was remarkably attenuated by NRG1 and stem cell treatment (Figure 2ciii, civ, cv and 2d). NRG1 injection achieved comparable beneficial effects as MSCe, but MSC-ERBB4 substantially outperformed NRG1 and MSCe, exhibiting a much smaller fibrotic area (Figure 2d, *P*<0.05 *versus* MI+NRG1, *P*<0.05 *versus* MI+MSCe), and more effectively preserved LV wall thickness (Figure 2e, *P*<0.05 *versus* MI+NRG1, *P*<0.05 *versus* MI+MSCe).

### Overexpressing ERBB4 potentiates MSC survival by effective homing and activation of the PI3K/Akt pathway

**Figure 3:**
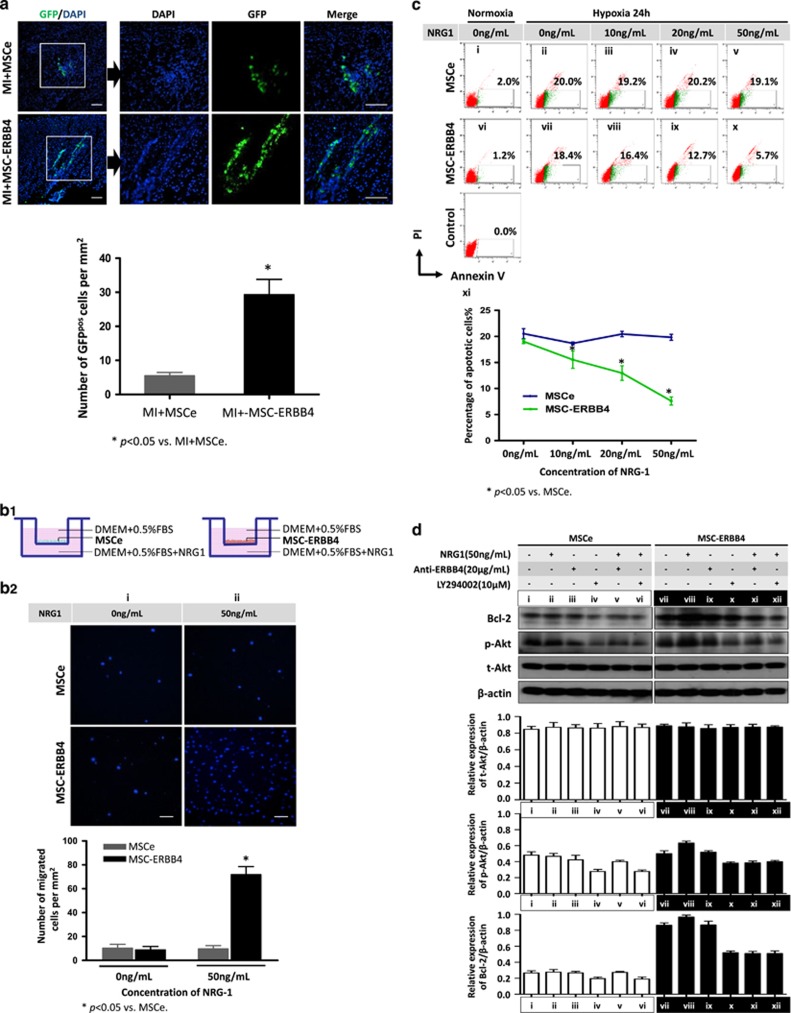
ERBB4 overexpression enhanced MSC survival through enhancing the mobility and anti-apoptosis by activating the PI3K/Akt pathway. **(a)** Surviving MSCe and MSC-ERBB4 were detected using anti-GFP antibody at 4 weeks post-MI. Representative images are shown. Quantification revealed that ERBB4 overexpression significantly improved MSC survival. Scale bar=200 *μ*m. *n*=12 for each group. (**b)** The NRG1-ERBB4 pathway enhanced MSC mobility. (**b1**) Experiment setting: MSCe or MSC-ERBB4 were seeded in the upper chamber of a transwell unit, with NRG1 added to the lower chamber to serve as an attractor. (**b2**) After hypoxic exposure for 6 hours, MSCe and MSC-ERBB4 exhibited equivalent movements (**b2i,** NS); however, MSC-ERBB4 demonstrated more aggressive mobility in the presence of NRG1 than MSCe did (**b2ii,** *P*<0.05 *versus* MSCe). Scale bar=200 *μ*m. (**c)** Annexin V/PI staining followed by flow cytometry were conducted to study the dynamic apoptotic rates of MSCe and MSC-ERBB4. Under hypoxia challenge, MSCe stayed incooperative to NRG1 (**cii**–**cv**), but the apoptotic rate of MSC-ERBB4 was attenuated by NRG1 in a dose-dependent manner under hypoxic condition (**cvii–cx**). The experiment was repeated three times and statistical analysis was performed (**cxi**). (**d)** Overexpressing ERBB4 in MSC reduced hypoxia-related apoptosis through the PI3K/Akt pathway. Additional NRG1 increased p-Akt accumulation in MSC-ERBB4 (**dviii** *versus* **vii**), but not in MSCe (**dii** *versus* **di**), and this effect could be partially blocked either by PI3K/Akt inhibitor LY294002 (**dxii** *versus* **dviii**), or anti-ER4 antibody (**dxi** *versus* **dviii**). The same trend was exhibited in Bcl-2 expression. The aforementioned western blotting was repeated three times and representative images are shown. Bcl-2, B-cell lymphoma 2; p-Akt, phosphorylated Akt; t-Akt, total Akt; anti-ERBB4, anti-ERBB4 antibody

Given that effective cell retention is essential for the beneficial effect of MSCs, we examined whether ERBB4 overexpression could enhance MSC survival following MI. As both MSCe and MSC-ERBB4 were successfully labeled with a GFP reporter, cell tracking was achieved using anti-GFP antibodies post-engraftment. Four weeks after cell injection, immunostaining with anti-GFP revealed that MSCs were still detectable (Figure 3a). As expected, significantly more GFP-positive cells were identified in the MSC-ERBB4 group than in the MSCe (Figure 3a, *P*<0.05 *versus* MSCe), demonstrating an ~2.5-fold improvement in cell survival.

Hypoxia in the ischemic area of the post-MI heart is thought to be the main cause of death of the transplanted MSCs.^25^ We used a reduced-serum medium containing 0.5% fetal bovine serum (FBS) and hypoxic culture (1% O_2_, 4% CO_2_) to treat MSCs with the intention of reproducing oxygen-glucose-deprived conditions *in vivo*. Abundant evidence supports fast mobilization and retention of MSCs at sites of injury following systemic or local intra-tissue infusion in the MI model.^26, 27, 28^ We thus determined whether ERBB4 overexpression would equip MSCs with superior mobility. After 6 hours hypoxic exposure in a transwell culture system, MSCe and MSC-ERBB4 demonstrated almost equivalent movement (Figure 3b2i, NS). Nonetheless in the presence of NRG1, notably enhanced migration was observed in MSC-ERBB4 towards its ligand NRG1, whereas MSCe did not readily react to the NRG1 treatment (Figure 3b2ii, *P*<0.05), indicating that the NRG1-ERBB4 pathway favorably affected cell mobility. It is relevant to guarantee a better MSC homing *in vivo*, as NRG1 expression is still detectable in MI hearts.^19^

The anti-apoptosis potential of MSC-ERBB4 was investigated using Annexin V/PI (propidium iodide) based flow cytometry analysis. No significant apoptosis was detected in cells cultured in normal conditions (normoxia, 15% FBS; Figure 3ci and cvi). Nevertheless, both MSCe and MSC-ERBB4 demonstrated a sensitive response to oxygen-nutrition deprivation (hypoxia, 0.5% FBS) by exhibiting an increased Annexin V positive population up to ~20% (Figure 3cii and cvii). To examine the role of NRG1-ERBB4 in cell apoptosis, a gradient concentration of NRG1 was added. In contrast to MSCe that stayed unresponsive to NRG1 (Figure 3ciii to cv), the apoptotic rate of MSC-ERBB4 was attenuated in response to NRG1 treatment administered in a dose-dependent manner, with the most significant inhibition apparent when 50 ng/ml NRG1 was applied (Figure 3cviii to cx and cxi). These results demonstrated that ERBB4 expression was essential in the response of MSC to the NRG1-mediated anti-apoptotic effect.

The mechanism involved in ERBB4-mediated cell protection against hypoxia is likely regulated by activation of the PI3K/Akt pathway.^29^ The activation of Akt refers to survival signals produced by the altered expression of anti-apoptotic protein, such as Bcl-2, or phosphorylating pro-apoptotic protein, such as Bad (Bcl-2-associated death promoter). We investigated whether the mechanism of cytoprotection driven by NRG1-ERBB4 signaling correlated with the PI3K/Akt pathway. The expression of total Akt (t-Akt), phosphorylated Akt (p-Akt), and Bcl-2 were examined by western blotting. In MSCe, the intensity of p-Akt was weaker in the cells treated with the Akt inhibitor LY294002 than in those that were untreated (Figure 3div versus di), suggesting that the Akt inhibitor effectively inhibited activation of Akt. We determined that Akt activity was not reliant on NRG1, because solely NRG1 treatment in MSCe did not increase p-Akt compared with those left untreated (Figure 3dii versus di). In addition, the combination of NRG1 and LY294002 did not alter the expression level of p-Akt (Figure 3div versus dvi). Regarding MSC-ERBB4, we observed that NRG1 treatment induced accumulation of p-Akt (Figure 3dviii versus dvii), and this phosphorylation of Akt by NRG1 was nullified by an additional Akt inhibitor LY294002 (Figure 3dxii versus dviii). In addition, the amount of upregulated p-Akt was remarkably decreased when ERBB4 expression was neutralized by anti-ERBB4 antibody treatment (Figure 3dxi versus dviii), indicating that NRG1-induced PI3K/Akt activation occurred through ERBB4 expression in MSCs. The same expression pattern to p-Akt was observed with Bcl-2. The data obtained in the MSC-ERBB4 suggested that the NRG1-ERBB4 pathway functioned through phosphorylated activation of Akt that further upregulated Bcl-2 expression and thereby protected cells from apoptosis.

### Activation of NRG1-ERBB4 signaling potentiates MSC-mediated myocardial repairs

**Figure 4:**
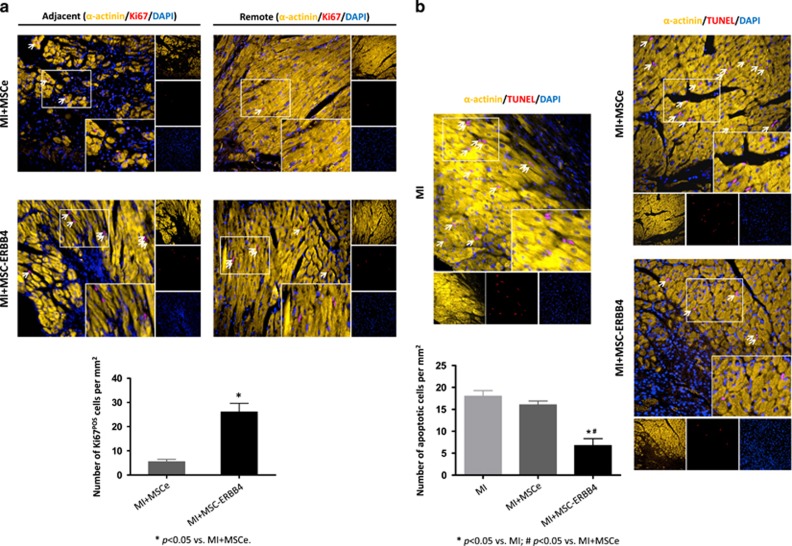
ERBB4 overexpression in MSCs provoked cardiomyocyte division and reduced cardiomyocyte loss. (**a**) Mature cardiomyocyte marker *α*-actinin and proliferation marker Ki67 indicated that cardiomyocytes underwent mitosis. Both MSCe and MSC-ERBB4 transplantation-stimulated cardiomyocyte proliferation in remote and adjacent areas. Representative images for each group are shown. Quantification indicated that MSC-ERBB4 induced more cardiomyocyte division than MSCe. Scale bar=200 *μ*m. *n*=8 for each group. (**b**) TUNEL-labeled apoptotic cardiomyocytes at 48 h after MI. Representative images are shown. MSC-ERBB4 transplantation provided better protection of cardiomyocytes against apoptosis than MSCe did. *n*=8 for each group

An intriguing role of the NRG1-ERBB4 pathway with respect to cardiac regenerative medicine is that NRG1 induces mature cardiomyocytes to re-proliferate.^11^ We consequently performed double immunostaining against *α*-actinin, a marker of mature cardiomyocytes, and Ki67, a marker located in the nucleus that indicates proliferative status. In accordance with previous findings, very few cardiomyocytes underwent proliferation under physiological conditions (Sham group, data not shown).^30^ No Ki67-positive cardiomyocytes were identified at various time points (24 h, 48 h, 7 days, 14 days, 28 days) in adult mice following infarction (MI group, data not shown). Nonetheless after stem cell transplantation, very small numbers of *α*-actinin and Ki67 double-positive cells were identified in both the MSCe group and MSC-ERBB4 group after infarction (Figure 4a). More Ki67-positive cells in the MSC-ERBB4 group than MSCe group were also observed, in both MI adjacent and remote region (Figure 4a, *P*<0.05).

The balance between cell proliferation and apoptosis is regarded as determinant in tissue regeneration. We next compared the apoptotic cell death among groups at 48 h after MI. As shown in Figure 4b, TUNEL (terminal deoxynucleotidyl transferase dUTP nick-end labeling) positive cardiomyocytes was reduced in MSCe treated group, but a more significant decrease was observed in the MSC-ERBB4 treated group (Figure 4b, *P*<0.05).

**Figure 5:**
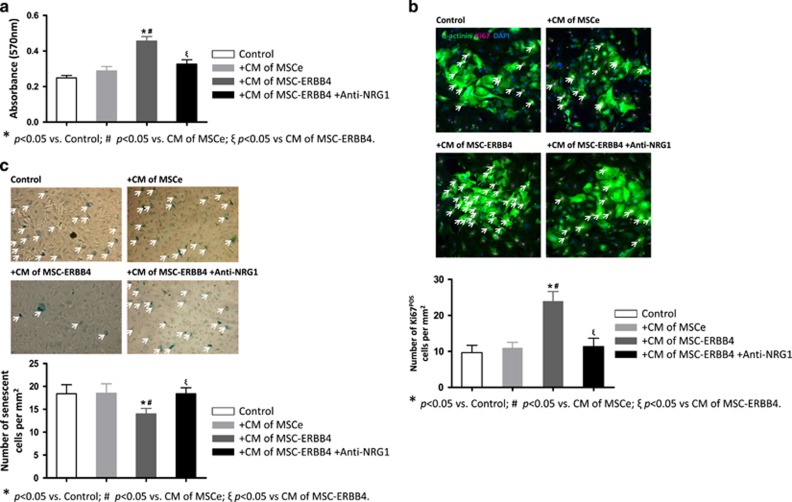

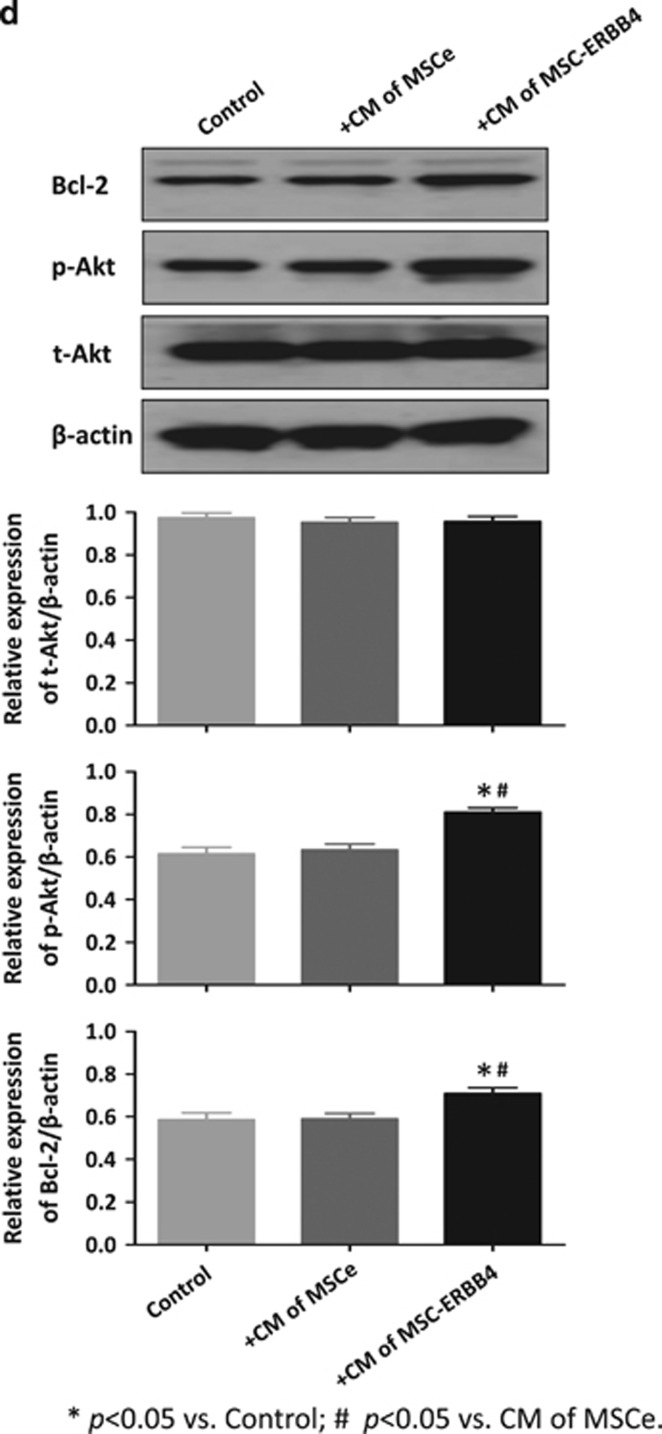
Conditioned medium of MSC-ERBB4 protects cardiomyocytes. (**a**) The conditioned medium of MSC-ERBB4 promoted cardiomyocyte growth, which was blunted by anti-NRG1 antibody. (**b**) More Ki67^pos^ cells were found in *α*-actinin^pos^ cardiomyocytes cultured with conditioned medium of MSC-ERBB4 than that of MSCe, indicating a more prosper cell dividing. The number of Ki67^pos^ cells declined when anti-NRG1 antibody was applied into conditioned medium of MSC-ERBB4. Scale bar=200 *μ*m. (**c**) The conditioned medium of MSC-ERBB4 prevented cardiomyocytes from senescence, which was erased by anti-NRG1 antibody. (**d**) The conditioned medium of MSC-ERBB4 prevented cardiomyocytes from apoptosis by enhancing p-Akt and Bcl-2 expression. DAPI, 4',6-diamidino-2-phenylindole; CM, conditioned medium; Bcl-2, B-cell lymphoma 2; p-Akt, phosphorylated Akt; t-Akt, total Akt; anti-NRG1, anti-NRG1 antibody

Cardiomyocytes can be induced to proliferate by activating NRG-ERBB4 signaling and, thus, produce global improvements in cardiac function.^11, 14^ We have demonstrated that MSC-ERBB4 injection induces cardiomyocyte proliferation and anti-apoptosis (Figure 4a and b). To seek *in vitro* evidence to support this finding, we investigated the possibility of MSC-ERBB4-mediated cardioprotection through three processes: cell proliferation, senescence and apoptosis. As the surface area of a 96-well transwell chamber (0.143 cm^2^ per well) might not have been sufficient or supportive for cardiomyocyte growth and immunostaining might be too obscure to present on the transwell membrane, we used concentrated conditioned medium collected from MSCe and MSC-ERBB4 following exposure to hypoxia for 24 h and evaluated their effects on cardiomyocytes, respectively. The primary neonatal cardiomyocytes, characterized by expression of the cardiac marker *α*-actinin (Supplementary Figure 3), were treated with conditioned medium and then subjected to hypoxic stress. Twenty-four hours later, MTT (3-(4,5-dimethylthiazolyl-2)-2, 5-diphenyltetrazolium bromide) assay was conducted and revealed distinct cell growth of cardiomyocytes supplemented with the conditioned medium of MSC-ERBB4, compared with that of MSCe (Figure 5a, *P*<0.05 *versus* MSCe). In accordance with the MTT results, more Ki67-positive cells, a marker indicating active phases of the cell cycle, were found in *α*-actinin positive cardiomyocytes cultured in the conditioned medium of MSC-ERBB4, compared with that of MSCe (Figure 5b, *P*<0.05 *versus* MSCe). The conditioned medium of MSC-ERBB4 also more efficiently protected cardiomyocytes against senescence than the conditioned medium of MSCe (Figure 5c, *P*<0.05 *versus* MSCe). The conditioned medium of MSC-ERBB4 protected cardiomyocytes against apoptosis by activating p-Akt and increasing anti-apoptotic protein Bcl-2 (Figure 5d). These data collectively suggested that the genetic manipulation of ERBB4 induced beneficial secretome in conditioned medium, through which cardiomyocytes were protected from hypoxic stress.

### Overexpressing ERBB4 in MSCs unexpectedly upregulated its ligand NRG1 to form an NRG1-ERBB4 autocrine loop

**Figure 6:**
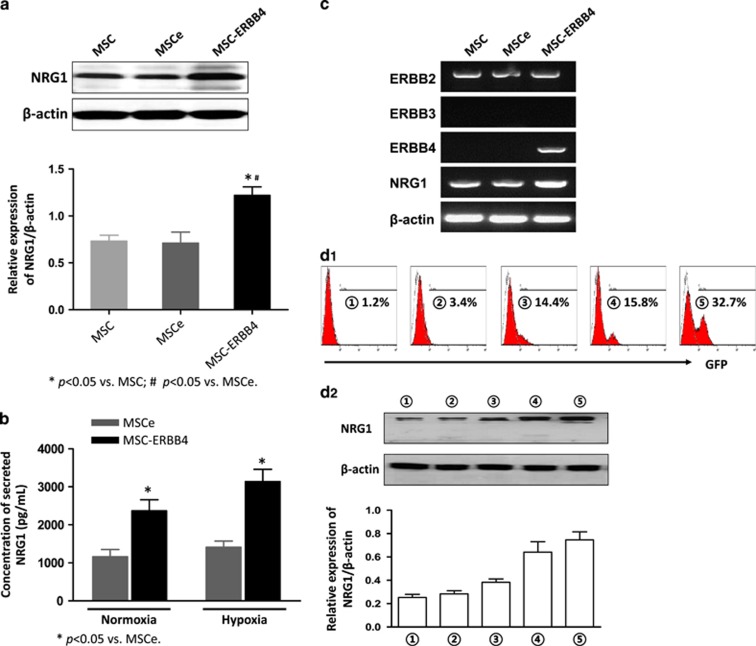

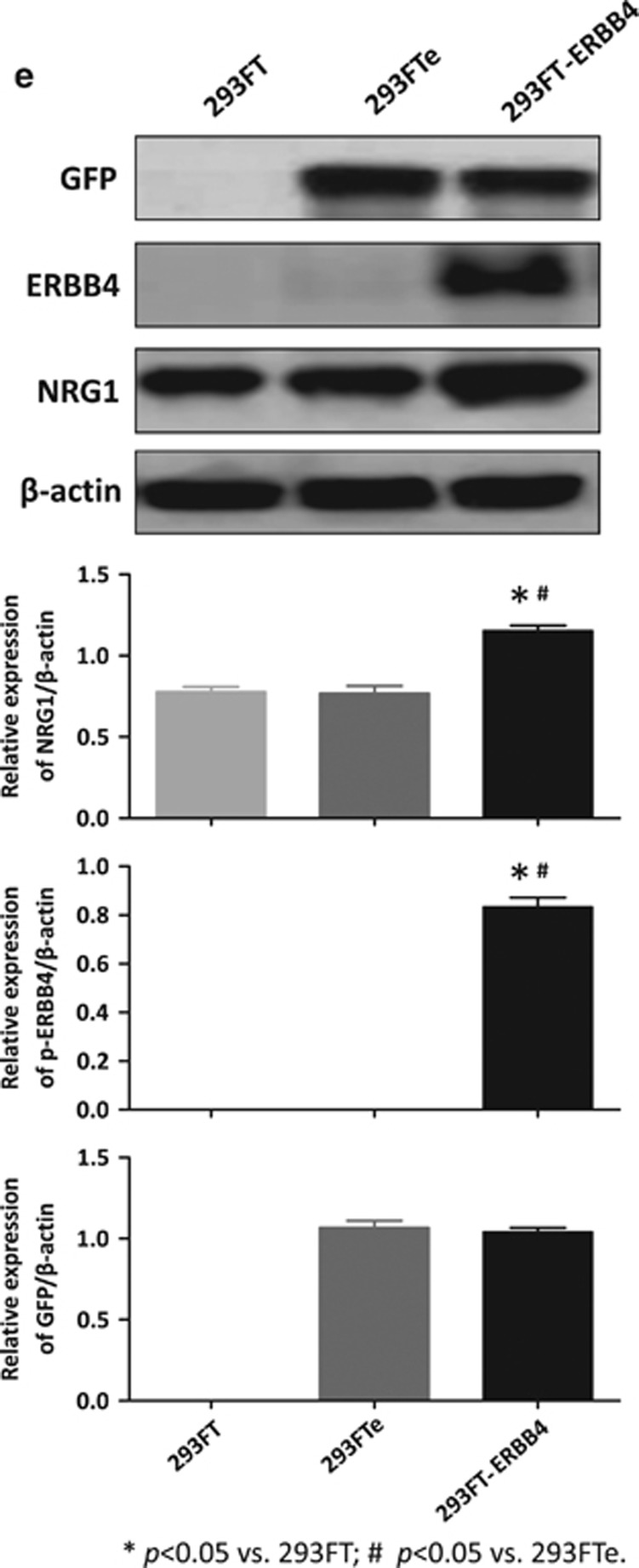
Overexpression of ERBB4 in MSCs unexpectedly activates a novel NRG1-ERBB4-NRG1 autocrine loop. Overexpressing ERBB4 in MSCs unexpectedly upregulated the synthesize and secretion of its ligand NRG1, detected by conducting western blotting (**a**) and ELISA (**b**), respectively. (**c**) Overexpressing ERBB4 in MSCs upregulated NRG1, but did not alter expression of ERBB2. ERBB3 remained negative before and after ERBB4 overexpressing. (**d**) Multiple transient transfections using pER4-GFP confirmed that upregulated NRG1 was attributed to exogenous ERBB4 overexpression. (**d1**) With increased transfection frequency shown by encircular numbers (**d1**
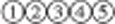
), GFP signal, which indicated transfection efficiency, was escalating accordingly, from 1.2% for the first time (**d1**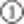) to 32.7% for the fifth time (**d1**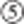). (**d2**) The expression of NRG1 was upregulated along with increased transfection efficiency. (**e**) In human 293FT cells, NRG1 expression was upregulated after ERBB4 transduction. The aforementioned experiments were repeated three times and representative images are shown. 293FTe, 293FT expressing pGFP; 293FT-ERBB4, 293FT expressing pER4-GFP

We examined NRG1 synthesis and secretion level in MSCe and MSC-ERBB4 by performing western blotting and enzyme-linked immunosorbent assay (ELISA) assays. Unexpectedly, we observed that the NRG1 level was significantly elevated in MSC-ERBB4, but not in MSCe, both in cellular (Figure 6a, *P*<0.05 *versus* MSCe) and concentrated conditioned medium (Figure 6b, *P*<0.05 *versus* MSCe). Hypoxia treatment further enhanced the NRG1 protein level in MSC-ERBB4 (Figure 6b, *P*<0.05 *versus* MSCe). ERBB4 manipulation upregulated NRG1, but did not alter the expression pattern of other ERBB family members (Figure 6c). Increased NRG1 expression caused by ERBB4 expressed in MSCs indicated that an NRG1-ERBB4-NRG1 autocrine loop existed. To test this hypothesis, we performed multiple transient transfection experiments using pER4-GFP plasmid, by which ERBB4 expression in MSCs can be reported and quantified using the population of GFP-positive MSCs in flow cytometry analysis. The NRG1 protein level among ERBB4 transfected cells was examined using western blotting. We determined that a gradual increase in the NRG1 level (Figure 6d1) was associated with an increasing number of GFP-positive cells (Figure 6d2). The increased expression of ERBB4 contributed to upregulation of NRG1, thus forming an NRG1-ERBB4-NRG1 autocrine loop. To confirm the intrinsic coupling of ERBB4 and NRG1, we performed another transfection experiment using human stromal 293FT cells. We detected an upregulated NRG1 protein level after the overexpression of ERBB4 (Figure 6e), suggesting that the NRG1-ERBB4-NRG1 loop indeed is conservative in mouse and human species.

### Transplantation of MSC-ERBB4 restores declined NRG1 in infarction region

**Figure 7:**
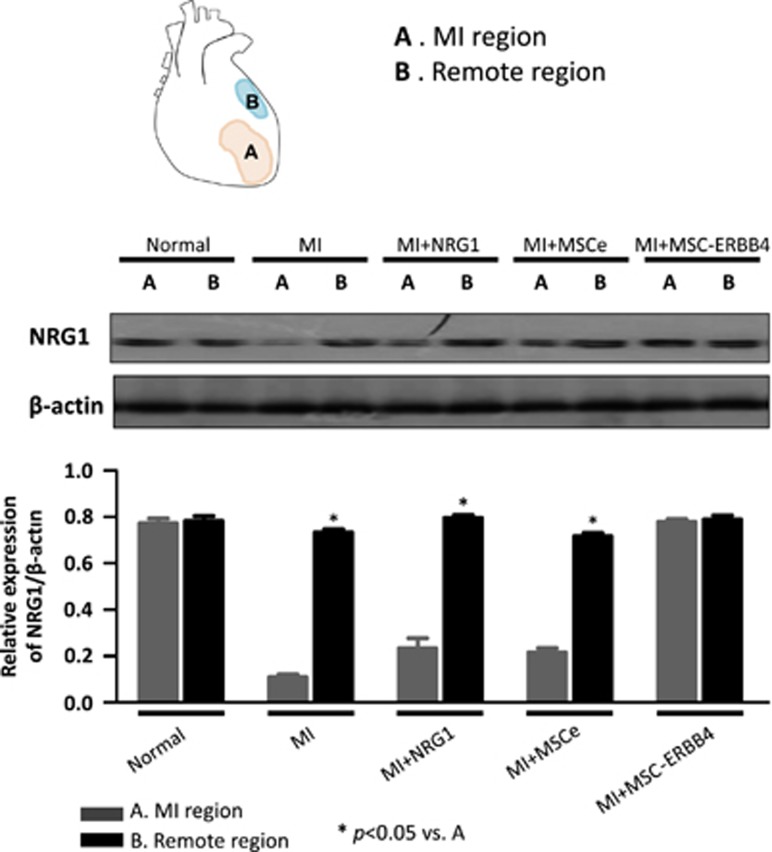
Transplantation of MSC-ERBB4 restores reduced NRG1 in MI region. The model of MI was achieved by LAD ligation, resulting in decreasing NRG1 in the infarct region. Neither MSCe nor NRG1 injection improved the situation, but MSC-ERBB4 transplantation restored NRG1 to a level comparable with that of the normal myocardium

Considering the cardiomyocyte proliferation induced by MSC-ERBB4 injection together with the documented roles of NRG1-ERBB4 signaling in stimulating cardiomyocyte re-proliferation, we examined whether transplantation of MSC-ERBB4 could restore declined NRG1 in infarction region. As expected, significantly decreased level of NRG1 was detected in the infarcted site compared with that of the remote non-infarcted site (Figure 7). However, the transplantation of MSC-ERBB4 could restore declining NRG1 level post-MI, but neither MSCe nor NRG1 (Figure 7).

**Figure 8:**
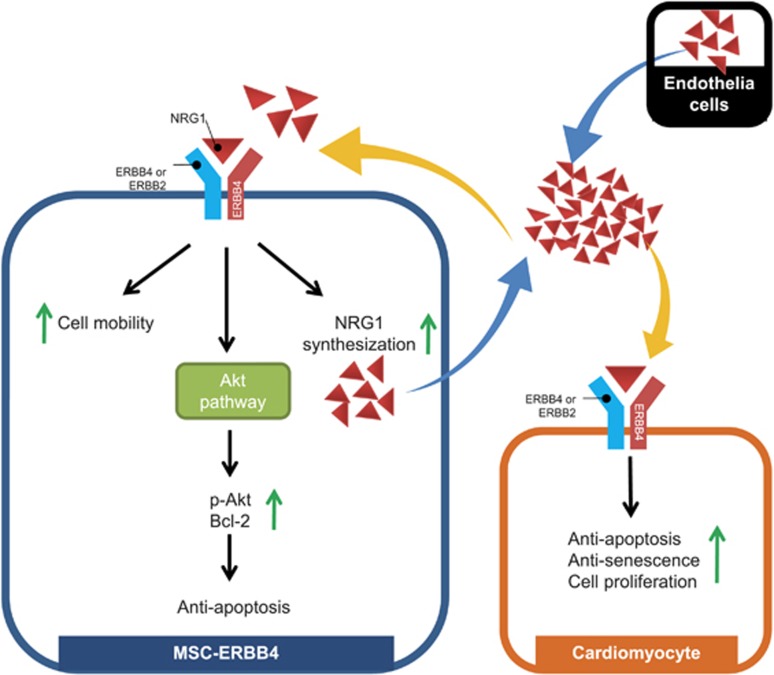
Schematic diagram of the NRG1-ERBB4-NRG1 autocrine pathway. Genetic manipulation of ERBB4 into MSC provides irreplaceable partner to ERBB2, the latter of which forms an ERBB2/ERBB4 heterodimer that binds to its ligand NRG1. ERBB4 can bind to NRG1 by forming a homodimer. Activation of the NRG1-ERBB pathway in MSC-ERBB4 enhances cell mobility and anti-apoptosis through the phosphorylation of Akt. Overexpressing ERBB4 in turn regulates the synthesis and secretion of NRG1. The released NRG1 contributes to restore the declined NRG1 level in the infarcted region and support cell growth, dividing and anti-apoptosis of cardiomyocytes. By ERBB4 overexpression in MSCs, we figured out a novel approach that benefits both MSCs and cardiomyocytes, and enable an effective myocardial repair after ischemia

As the activation of NRG1-ERBB4 autocrine loop leaded to increasing NRG1 level (Figure 6), we intended to examine the role of self-activated NRG1 on the cardiomyocytes. We introduced anti-NRG1 antibody to neutralize the soluble NRG1 in the concentrated conditioned medium of MSC-ERBB4. The application dosage (10 *μ*g/mL) was provided far beyond sufficient, according to ELISA results (3000 pg/mL in conditioned medium of MSC-ERBB4, Figure 6b), to allow an effective inhibition. When anti-NRG1 was applied, the number of proliferating cardiomyocytes declined to a level comparable with that in conditioned medium of MSCe (Figure 5a and b), suggesting that the NRG1 in the MSC-ERBB4 conditioned medium indeed was responsible for stimulating cardiomyocytes proliferation. The conditioned medium of MSC-ERBB4 protected cardiomyocytes from senescence, but anti-NRG1 antibody erased this effect (Figure 5c). These data collectively indicated that the transplantation of MSC-ERBB4 might serve as an NRG1 factory to restore declined NRG1 in infarction region and regenerate cardiomyocytes (Figure 8).

## Discussion

In this study, we demonstrated that introducing ERBB4 into MSCs not only ameliorated MSC survival potential, but also stimulated cardiomyocyte regeneration. Most importantly, for the first time, we identified a positive-feedback autocrine loop in the NRG1-ERBB4 signaling pathway. ERBB4 overexpression potentiated MSC survival in the infarcted heart, and enhanced NRG1 generation to restore the declining NRG1 in the infarcted region and stimulate cardiomyocyte division.

Current evidence supports the essential roles of NRG1 and ERBB family in myocardial physiology. In mice that lacked functional NRG1, ERBB2 or ERBB4 signaling, the primitive ventricles failed to undergo expansion and trabeculation, leading to abnormally thin myocardium and enlarged ventricles that cause embryonic lethality at E10.5.^31, 32^ In adult hearts, NRG1-ERBB4 signaling modulates cell growth, survival, sarcomere structure and the re-entry of cardiomyocytes into the cell cycle.^11^ These data collectively suggest that the NRG1-ERBB pathway has a vital role in the heart under adverse physiological and pathological circumstances. Increasing the activity of the NRG1-ERBB signaling complex might provide a viable strategy for treating heart failure.

By transplanting MSC-ERBB4 into the infarcted myocardium, we established an intersection that benefits both MSCs and host myocytes and built a reciprocal relationship between the cells, either endogenous or exogenous, and the surrounding milieu (Figure 8): shows cells in the damaged region release cytokines that attract the infused MSC-ERBB4,^33, 34^ and NRG1 may be involved in the chemotaxis process; the presence of NRG1 in the infarcted region improved MSC-ERBB4 cell survival by attenuating apoptosis via PI3K/Akt pathway; MSC-ERBB4 upregulates the synthesis and secretion of its ligand NRG1, the latter of which stimulates cardiomyocyte cell cycle re-entry and a rebound in NRG1 level in the infarcted myocardium. MSC-ERBB4 may act as a moving ‘NRG1 factory' in an autocrine and paracrine manner to activate survival signaling in MSCs and the host cardimyocytes. It should be noted that although ERBB4 overexpression leads to NRG1 secretion, the self-activated NRG1 is not sufficient to alter the cell behavior. Cell mobility and anti-apoptosis of MSC-ERBB4 differed little to that of MSCe unless additional NRG1 was provided (Figure 3bii and 3c), suggesting that this autocrine loop is likely autonomic, and thus reduces the risk of malignancy (Figure 1d).

In summary, ERBB4 overexpression potentiates MSC survival in the infarcted heart, enhances NRG1 generation to restore the declining NRG1 in the infarcted region and stimulates cardiomyocyte division. ERBB4 overexpression in MSCs may provide a novel molecular strategy for myocardial repair and regeneration.

## Materials and Methods

An expanded Materials and Methods section is included in the Supplementary Materials.

MSCs were isolated and cultured as previously described.^35^ Characterized MSC at passage 4–8 were used in the study. Expression profile of NRG1-ERBB signaling was examined by RT-PCR. MSC were infected with lentivirus containing ERBB4-GFP (pER4-GFP) or GFP (pGFP) and stably transduced. The efficiency of gene delivery was confirmed by RT-PCR, western blotting (ERBB4 (Abcam, Cambridge, UK; ab19391); p-ERBB4 (Abcam, ab109273); *β*-actin (Santa Cruz, Dallas, TX, USA; SC-47778)) and cell fluorescence before further transplantation into the infarcted hearts or *in vitro* experiments. Tri-lineage differentiation capability was examined after genetic modification. Tumor formation was examined by subcutaneously transplanting 1 × 10^6^ MSCe or MSC-ERBB4 into NOD-SCID mice, with mESCs inoculated to the other side of the animal as controls (*n*=4). Tumorigenesis in mice was kept under observation for 8 weeks. A MI model was constructed by permanent ligation of the LAD in female C57/B6 mice (6–8 weeks) as described previously.^36, 37^ Immediately following LAD ligation, NRG1 (Abm, Richmond, BC, Canada; RP155006, 100 *μ*g/kg), or 3 × 10^5^ transduced MSCs suspended in 30 *μ*L PBS were injected at 5 sites in the anterior and posterior infarct border zones of the ischemic myocardium. Animal care and all experimental procedures adhered to the Guidelines for the Care and Use of Laboratory Animals prepared by the National Academy of Sciences and published by the National Institutes of Health, and were approved by the Committee on the Use of Live Animals in Teaching and Research at the University of Hong Kong. Invasive cardiac hemodynamic assessment was performed 4 weeks after MI to measure the cardiac function. Masson trichrome staining (KeyGEN, Nanjing, China; KGMST-8003) was used to determine cardiac remodeling. Immunofluorescence staining using anti-GFP antibody (Santa Cruz, SC-8334) was performed to determine cell survival *in vivo.* Double staining using Ki67 (Abcam, ab15580), a cell proliferation marker, and *α*-actinin (Sigma, St. Louis, MO, USA; A7811), a mature cardiomyocyte marker, was performed to determine cardiomyocytes in the active cell cycle phase. TUNEL (Keygen, KGA7062) and *α*-actinin double staining was performed to quantify apoptotic cardiomyocytes at 48 h post-MI *in vivo*. Cultured MSCs, with or without ERBB4 expression, were challenged with hypoxia (1% O_2_, 4% CO_2_). Cell mobility and anti-apoptosis were analyzed using transwell assay and Annexin V/PI staining (eBioscience, San Diego, CA, USA; 88-8007-74), respectively. Western blotting against p-Akt (Cell-Signaling, Boston, MA, USA; 9611) Bcl-2 (Cell-Signaling, 2870) were used to determine the mechanism involved in anti-apoptotic effects. To test the protective effect of paracrine factors secreted by MSCs against hypoxia-induced cell death, conditioned medium from MSC hypoxic cultures was added to cardiomyocytes. MTT, Ki67 staining, cell senescence-associated *β*-galactosidase staining (Millipore, Boston, MA, USA; KAA002) and western blotting against p-Akt was conducted, to evaluate cell growth, proliferation, anti-aging and anti-apoptosis effects, respectively. ELISA (USCN, Houston, TX, USA; E91866Mu) and western blotting against NRG1 (Santa Cruz, SC-348) were used to assess the expression after ERBB4 overexpression in MSCs. RT-PCR was performed to measure the changes of NRG1-ERBB signaling after ERBB4 overexpression in MSCs. To verify an autocrine loop in the NRG1-ERBB4-NRG1 pathway, a multiple transient transfection using pER4-GFP was performed, in which transfection efficiency was detected by GFP fluorescence and NRG1 expression was detected by western blotting. To validate the role of self-activated NRG1 on cardiomyocytes, anti-NRG1 antibody (Santa Cruz, SC-348, 10 *μ*g/mL) was applied to the conditioned medium of MSC-ERBB4, and cell growth, proliferation and senescence were evaluated. western Blotting was performed to assess NRG1 level in the infarcted myocardium and remote region after NRG1 and stem cell injection. Student's *t*-test or one or two-way ANOVA with the Bonferroni *post hoc* test were applied to determine the statistical significance (*P*<0.05).

## Figures and Tables

**Table 1:** Summary of cardiac functional parameter

	**HR (per min)**	**ESP (mm Hg)**	**EDP (mm Hg)**	**+dp/dt (mm Hg/s)**	**−dp/dt (mm Hg/s)**
Sham	201.20±46.67	75.17±7.20	5.14±5.22	2891.31±215.27	−2327.65±259.57
MI	228.90±43.74	52.47±13.42^a^	5.60±9.56	1126.19±229.84^a^	−669.05±213.08^a^
MI+NRG1	201.43±38.92	62.58±18.46^b^	6.45±8.13	1607.62±239.19^a,b^	−1201.64±345.76^a,b^
MI+MSCe	193.77±28.72	74.32±15.87^b^	6.62±6.70	1637.91±±159.98^a,b^	−1318.61±530.07^a,b^
MI+MSC-ERBB4	193.81±45.05	75.25±10.35^b^	6.58±6.03	2199.95±143.42^a,b,c,d^	−1769.15±275.20^a,b,c,d^

Abbreviations: HR, heart rate; ESP, end-systolic pressure; EDP, end-diastolic pressure

^a^*P*<0.05 *versus* sham group

^b^*P*<0.05 *versus* MI group

^c^*P*<0.05 *versus* MI+NRG1 group

^d^*P*<0.05 *versus* MI+MSCe group

## Abbreviations

Akt: v-akt murine thymoma viral oncogene homolog 1
Bcl-2: B-cell lymphoma 2
ELISA: enzyme-linked immunosorbent assay
ERBB2: v-erb-b2 avian erythroblastic leukemia viral oncogene homolog 2
ERBB3: v-erb-b2 avian erythroblastic leukemia viral oncogene homolog 3
ERBB4, ER4: V-erb-b2 avian erythroblastic leukemia viral oncogene homolog 4
ESPVR: end-systolic pressure-volume relationship
FBS: fetal bovine serum
GFP: green fluorescent protein
IGF: insulin-like growth factor 1
LAD: left anterior descending artery
LV: left ventricular
mESCs: mouse embryonic stem cells
MI: myocardial infarction
MSC: mesenchymal stem cell
MSCe: MSCs expressing empty vector
MSC-ERBB4: MSCs expressing ERBB4
MTT: 3-(4, 5-dimethylthiazolyl-2)-2, 5-diphenyltetrazolium bromide
NRG1: neuregulin1
p-Akt: phosphorylated Akt
PI: propidium iodide
PV-loop: pressure-volume loop
RT-PCR: reverse transcription polymerase chain reaction
t-Akt: total Akt
TUNEL: terminal deoxynucleotidyl transferase dUTP nick-end labeling
