# Supplementary figures and images for: Activation of NRG1-ERBB4 signaling potentiates mesenchymal stem cell-mediated myocardial repairs following myocardial infarction
Source: Cell Death Dis. 2015 May 21;6(5):e1765–. doi: 10.1038/cddis.2015.91 (PMC4669719; doi:10.1038/cddis.2015.91)

**A****i. MSC**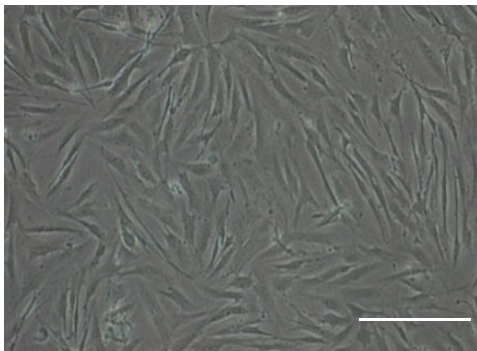**ii. adipogenesis**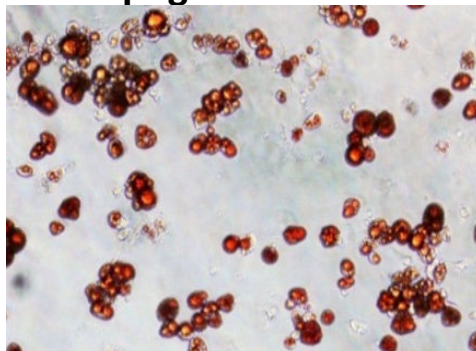**iii. chondrogenesis**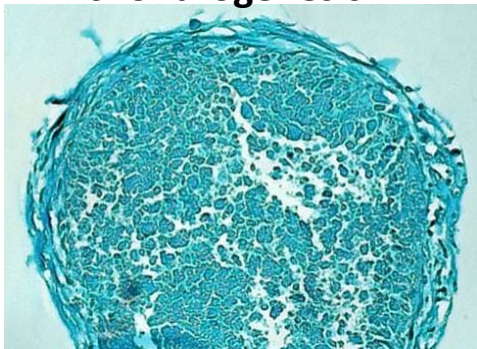**iv. osteogenesis**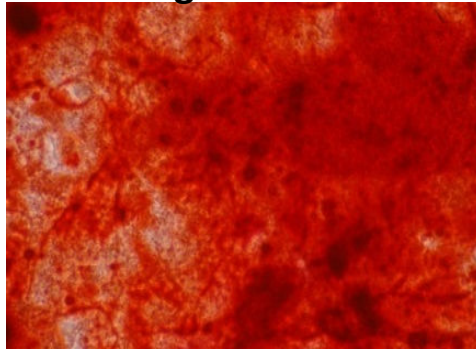**B**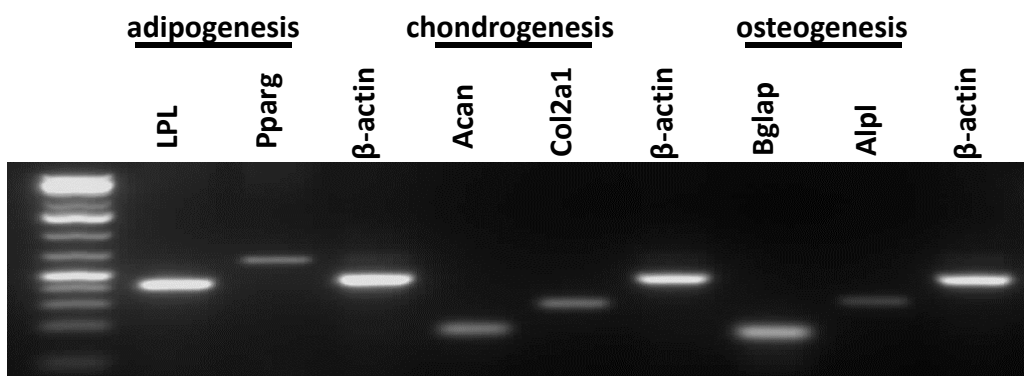**C**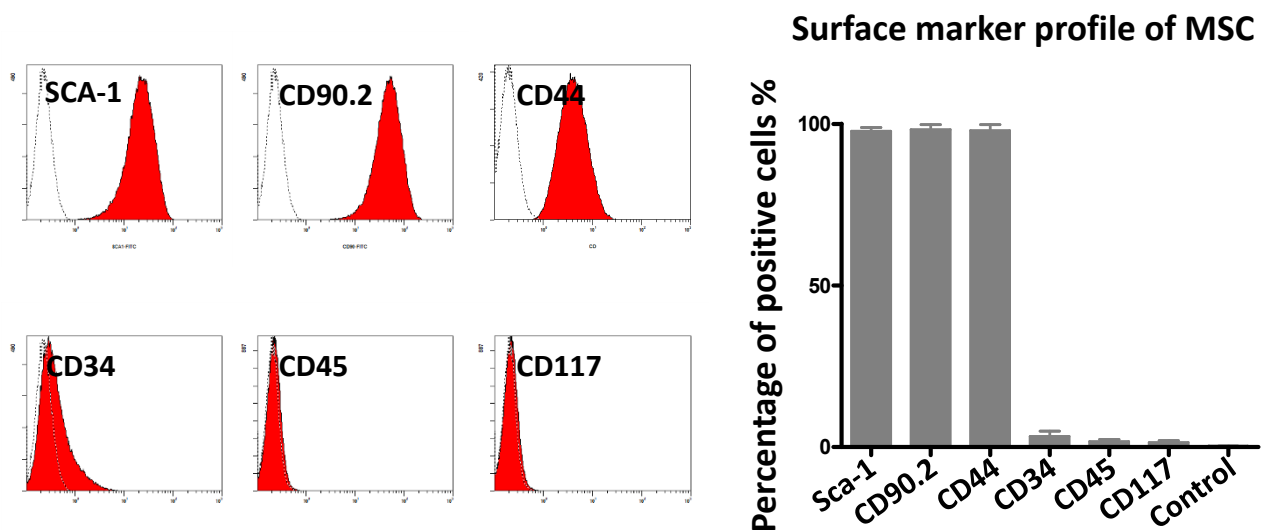

Supplementary Figure I

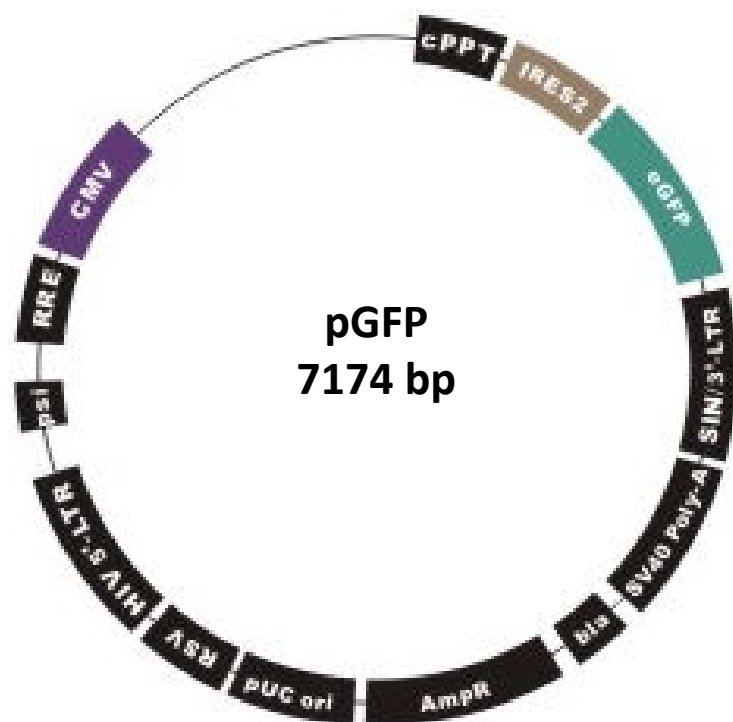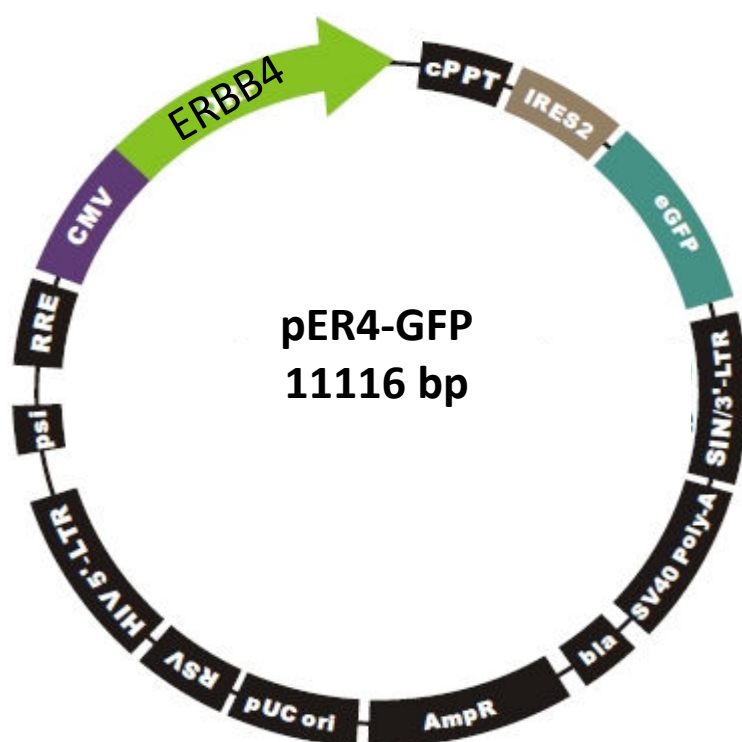

**A**

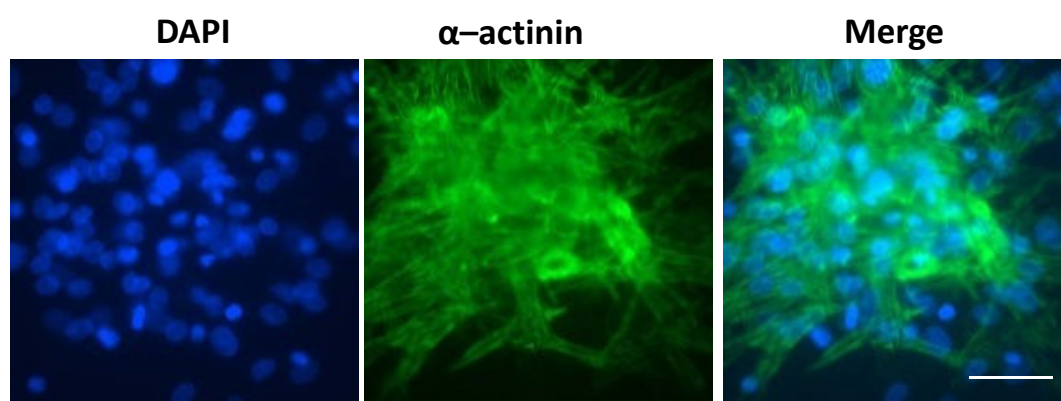

Supplement: Supplementary Figure 1 [file cddis201591x1.pdf]
