# Supplementary material for: Activation of NRG1-ERBB4 signaling potentiates mesenchymal stem cell-mediated myocardial repairs following myocardial infarction
Source: Cell Death Dis. 2015 May 21;6(5):e1765–. doi: 10.1038/cddis.2015.91 (PMC4669719; doi:10.1038/cddis.2015.91)
Supplement: Supplementary Information [file cddis201591x2.doc]

**Activation of NRG1-ERBB4 Signaling Potentiates Mesenchymal Stem Cell- mediated Myocardial Repairs Following Myocardial Infarction**

Xiaoting Liang, MSc;^1^ Yue Ding, M.Sc;^1,2^ Yuelin Zhang, PhD;^1^ Yuet-Hung Chai, BSc;^1, 2^ Jia He, MSc;^1^ Sin-Ming Chiu, M.Phil;^1^ Fei Gao, MSc;^1^ Hung-Fat Tse, MD, PhD;^1, 3-5^* Qizhou Lian, MD, PhD^1, 3-6^*.

^1^Department of Medicine, the University of Hong Kong, Hong Kong;

^2^Organ Transplantation Institute, Xiamen University, Fujian Province, PR China

^3^Research Centre of Heart, Brain, Hormone, and Healthy Aging, Li Ka Shing Faculty of Medicine, the University of Hong Kong, Hong Kong;

^4^Shenzhen Institutes of Research and Innovation, University of Hong Kong, China;

^5^Hong Kong-Guangdong Joint Laboratory on Stem Cell and Regenerative Medicine, China;

^6^Department of Ophthalmology, Li Ka Shing Faculty of Medicine, the University of Hong Kong, Hong Kong

**Running title:** NRG1-ERBB4-NRG1 loop in MSC-mediated cardiac repair

***Address correspondence to:**

Qizhou Lian, MD, PhD,

Cardiology Division,

Department of Medicine,

The University of Hong Kong,

Hong Kong.

Tel: +852-28315403

Fax: +852-28162095

Email:[qzlian@hku.hku.hk](mailto:qzlian@hku.hku.hk) (Q Lian); or [hftse@hkucc.hku.hk](mailto:hftse@hkucc.hku.hk) (HF Tse)

**This file includes:**

**1. Supplementary Materials and Methods**

**2. Supplementary Table I**

**3. Supplementary Figure Legends**

**4. Supplementary References**

**Supplemental Materials and Methods**

**Laboratory animals**

All protocols adhered to the Guidelines for the Care and Use of Laboratory Animals prepared by the National Academy of Sciences and published by the National Institutes of Health, and was approved by Committee on the Use of Live Animals in Teaching and Research at the University of Hong Kong.

**Isolation and Characterization of** **BM-MSCs**

MSCs were obtained from C57/B6N male mice (6~8 weeks). For mouse bone marrow MSCs isolation, tibias and fibulas of C57/B6N male mice were isolated and flushed by 29-G syringes. Then the cells were cultured in complete medium consisted of DMEM/low glucose (Hyclone, SH30021.02), 15% FBS (Life Technologies, 16000), 5 ng/mL epidermal growth factor (PeProTech, AF-100-15), 5 ng/mL fibroblast growth factor (PeProTech, 100-18B), 0.1mM 2-mercaptoethanol (Life Technologies, 21985023), Glutamax (Life Technologies, 35050061), NEAA (Life Technologies, 11140050) and Penicillin-Streptomycin (Life Technologies, 15140122). They were kept expanding till passage 4~8, then a subset of MSCs were used for characterization as described below. The others were stocked for transplantation and *in vitro* experiments.

MSCs were characterized by surface marker profiling and ability to differentiate into 3 germ layers. Cell surface antigens for MSCs were analyzed by flow cytometry. We incubated 1.5 x 10^5^ cells with each of the following conjugated antibodies: SCA1-FITC (eBioscience, 11-5981-82), CD90.2-FITC (eBioscience, 11-0903-82), CD44-APC (eBioscience, 17-0441-82), CD34-FITC (eBioscience, 11-0341-82), CD45-PE (eBioscience, 12-0451-82), CD117-PE (eBioscience, 12-1171-82). Nonspecific fluorescence was determined by incubation of similar cell aliquots with isotype-matched antibodies (eBioscience). Data were analyzed by collecting 30000 events on Beckman Coulter FC500 using CXP Analysis 2.0 software. For differentiation ability, adipogenesis, chondrogenesis, and osteogenesis of MSCs were performed by relative commercial kits (adipogenesis: Life Technologies, A1007001; chondrogenesis: Life Technologies, A1007101; osteogenesis: Life Technologies, A1007201) according to the manufacturer’s instruction. Oil Red, Alcian Blue and Alizarin Red staining for adipocytes, chondrocytes, and osteocytes, respectively, was performed using standard techniques after differentiation. Reverse transcription polymerase chain reaction (RT-PCR) was performed to determine the lineage specific gene expression after differentiation with primers listed in Supplementary Table I. Detail procedure was described as below.

**RT-PCR**

Total RNA was extracted using RNeasy kit (Qiagen, 74104). 1 µg of total RNA was used for the gene ration of first-strand cDNA using First-Strand cDNA Synthesis Kit (Takara, 6110) as per the manufacturer's recommendations. Lineage specific genes, including LPL, Pparg, Acan, Col2a1, Bglap and Alpl were determined as part of MSC characterization. ERBB2, ERBB3, ERBB4, and NRG1 were determined before and after ERBB4 overexpression in MSCs. β-actin was loaded as controls in the above mentioned reactions. Primers were listed in Supplementary Table I. PCR conditions were 36 cycles of 94°C for 1 minute 55°C for 30 seconds, 72°C for 1 minute. The amplified products were analyzed by electrophoresis in 2.0% agarose gel containing ethidium bromide.

**Lentivirus vector construction,** **virus packaging, infection and efficiency assessment**

Two lentivirus based recombinant plasmids were constructed by GeneCopoeia, China (<http://www.fulengen.com/index.php>) with map showed in Supplementary Figure II. One was inserted by ERBB4 together with GFP (pER4-GFP), with the other inserted by GFP only (pGFP) in same backbone served as a control. The structure of both plasmids contained GFP protein so as to readily determine the transfection and infection efficiency, and to provide a believable marker for *in vivo* cell tracking.

For virus packaging, 293FT cells were cultured in 10 cm plates until they were 80% confluent. We used the 3^rd^ generation lentiviral packaging system purchased from Addgene (http://www.addgene.org/), which contained 4 plasmids in total: 2 packaging plasmids (pMDLg/pRRE and pRSV-Rev), an envelope plasmid (pMD2.G), and a transfer plasmid (pER4-GFP or pGFP). As pER4-GFP was carrying a large ERBB4 insert (3879 bp), we modified the plasmid formula referring to the protocol published by al Yacoub, N et al.[^1^](#_ENREF_1) The practical formula for pGFP viral packaging was as follows: 36 μg pER4-GFP: 12 μg pMDLg/RRE: 18 µg pMD2.G: 6 μg pRSV-Rev. pGFP viral packaging formula was as follows: 10 μg pGFP: 6.52 μg pMDLg/RRE: 2.52 µg pMD2.G: 3.52 μg pRSV-Rev. 293FT cells were transfected by using lipofectamine2000 (Life Technologies, 11668019) according to the standard manual. After 48 hours, the supernatant containing virus was collected, cleared by centrifugation, filtered by 0.45 μm millipore filter, and then stored at -80° C in aliquots until use. The second supernatant was processed identically after additional 24 hours incubation. For infection, a final concentration of 8 μg/mL polybrene (Millipore, TR-1003-G) was added together with the viral supernatant into MSCs in passage 4 at 30%-40% confluence. Multi-infections were performed to achieve optimal gene transfer. All MSCs were expanded to passage 4~8 and then used for further studies. The efficiency of gene transduction was evaluated by immunofluorescence, RT-PCR and Western blotting.

**MI model**

Female C57/B6N mice aged 6~8 weeks were grouped to receive direct intramyocardial injections of NRG1 (Abm, RP155006), MSCe or MSC-ERBB4 immediately after left anterior descending artery (LAD) occlusion. The brief surgical protocol was as follows: Female mice were anaesthetized by intraperitoneal injection of Ketamine (100 mg/kg) and Xylazine (20 mg/kg), and connected to a mouse ventilator via orotracheal intubation. Then left thoracotomy was performed at 4^th^ intercostal space. The hearts were exposed and the LAD was ligated 2 mm from the origin of the aorta with 8-0 suture. Then the animals were randomized to receive direct intramyocardial injection of 1) PBS (MI group; N = 13); 2) 100 μg/kg NRG1 (MI + NRG1 group, N = 12); 3) 3 x 10^5^ MSCe (MI + MSCe group; N = 16; 4) 3 x 10^5^ MSC-ERBB4 (MI + MSC-ERBB4 group; N = 17) at three left ventricular (LV) sites near the infarct border zone. Another group of mice underwent thoracotomy without LAD ligation and served as controls (Sham group; N = 12). After final invasive hemodynamic assessments at 4 weeks, hearts were harvested and used for histological examinations.

**Invasive cardiac hemodynamic assessment**

At 4 weeks after surgery, animals were anesthetized and ventilated as described above before hemodynamic measurement. A 1.2-F pressure-volume (PV) conductance catheter connected to an ADVantage PV loop system (Scisence Inc) was inserted into the LV cavity via the right carotid artery. Hemodynamic parameters, including LV pressure and positive maximal (+dp/dt) and negative pressure derivative (-dp/dt), were recorded by LabScribe recording and analysis software (Scisence Inc). The slope of the end-systolic pressure-volume relationship (ESPVR) measurement was obtained before and after inferior vena cava occlusion to produce baseline and dynamic pressure-volume loops under changes in preload.[^2^](#_ENREF_2)

**Morphometric evaluation of infarct size**

To elucidate the severity of myocardial fibrosis, Masson trichrome staining (KeyGEN, KGMST-8003) was performed on paraffin sections from each tissue block. The infarct size was quantified by the average ratio of fibrosis area to the total LV area (percent fibrosis area). Infarct wall thickness was determined by the average of 3 equidistant measurements on each section.

**Immunostaining**

Mice tissue was paraformaldehyde fixed, paraffin embedded, and sectioned for standard immunostaining. To quantitatively study cell engraftment, tissues were stained with anti-GFP (Santa Cruz, SC-8334). To measure proliferating cardiomyocytes, tissues were double stained with α-actinin (Sigma, A7811) and Ki67 (Abcam, ab15580). Ten mice from each group were analyzed. Six sections were randomly collected from each mouse to calculate the average positive immunoactivity. Sections were analyzed with a deconvoluted fluorescent microscope and Metamorph software.

***In vivo* Apoptosis analysis**

*In vivo* TUNEL (Terminal deoxynucleotidyl transferase dUTP nick end labeling) assay (TRITC labeled) (Keygen, KGA7062) was applied to measure host myocyte apoptosis according to the manufacturer's instructions. Procedures were described as follows: Tissue sections were dewaxed and rehydrated according to standard protocols. Proteinase K working solution was prepared by diluting 50X Proteinase K in PBS. The slides were added Proteinase K working solution and incubated in 37 °C for 30 minutes, and then washed twice with PBS, 5 minutes each time. For each sample, 50 μL TdT reaction buffer was prepared by mixing 45 μL Equilibration Buffer, 1μL biotin-12-dUTP and 4 μL TdT Enzyme. For positive control: Before beginning the labeling procedure as described below, the samples were incubated with 100 μL DNase I Solution (3000U~5000U per sample) for 30 minutes at 37 °C to induce DNA strand degradation. For each sample, 50 μL TdT reaction buffer was applied. The slides were incubated in 37 °C for 60 minutes in dark. The slides were rinsed twice with PBS in dark, 5 minutes each time. For each sample, 50 μL Strptavidin-TRITC working solution was prepared by diluting 5 μL Strptavidin-TRITC with 45 μL Labeling buffer. For each sample, 50 μL Strptavidin-TRITC working solution was applied, and then the slides were incubated in 37 °C for 30 minutes in dark. After washing twice with PBS in dark, DAPI was added to the samples and incubated 10 minutes at room temperature in dark. After washing twice with PBS in dark, the slides were mounted and assayed with a fluorescence microscope using excitation wave 543 nm.

**Cell mobility-migration assay**

This experiment was to testify the genetic modification on MSCs enhances cell mobility. With the purpose to imitate a nutrition-deficient milieu *in vivo*, low FBS (0.5% FBS) medium and hypoxic culture (1% O_2_, 4% CO_2_) were applied. 2 x 10^4^ cells were seeded on the upper chamber of the transwell apparatus with pore size of 8 μm (Corning, #3422). In the lower chamber, we supplemented the medium with NRG1 (50 ng/mL) as attractant to evaluate the effect of NRG1-ERBB4 signal pathway on cell mobility. The experimental settings were illustrated in Figure 3, B1. Six hours later, the cells were fixed using 4% paraformaldehyde for 30 minutes. Non-migrated cells were scraped off the upper surface of the membrane with cotton swab. Migrated cells remaining on the bottom surface were stained with DAPI for 5 minutes and then proceeded with imaging under fluorescent microscope. For each group we have triplicate wells with 8 random sights each well.

***In vitro* Apoptosis analysis**

This experiment was performed to measure the effects of NRG1-ERBB4 signaling on cell apoptosis subjected to hypoxia stress. MSCe and MSC-ERBB4 were treated with gradient concentration of NRG1 (0 ng/mL, 10 ng/mL, 20 ng/mL, 50 ng/mL) and kept in culture under hypoxia for 24 hours. Cells were collected and examined with Annexin V/PI apoptosis detection kit (eBioscience, 88-8007-74) according to the manufacturer's instructions. Stained cells were analyzed by flow cytometry.

**Isolation and Characterization of mouse neonatal cardiomyocytes**

For mouse neonatal cardiomyocyte isolation, embryonic hearts were micro-dissected away from the embryo of C57/B6N mice and placed in a 35 mm dish containing pre-cooled HBSS. Then the tissues were cut into 1~3 mm^3^ pieces and transferred into a 50 mL conical tube containing 10~15 mL 0.05% Trypsin (Life Technologies, 25300062). The tube was slowly rotated for 15 minutes in a 37° C water bath. The supernatant was gently removed after 3 minutes standing. Fresh 0.05% trypsin was added and the tube was rotated at 37°C for another 15 minutes. Then the supernatant was gently collected into a new conical tube containing 10 mL complete Claycomb Medium (Sigma, 51800) to stop the enzymatic reaction. The digestion was repeated as described above for 2~5 times until the heart structures were reduced to a single matrix conglomerate. The collected cells were then placed in a 10 cm culture dish and incubated at 37° C for 2~3 h. After incubation, the supernatant containing predominantly myocytes was removed and spun for 10 minutes at 1000 rpm. The cells were then placed in a 6-well culture plate containing 2 mL/per well Claycomb Medium at 37 °C. The cells were split into culture plates when they reached confluence according to different experimental designs (MTT: 96-well plate; immunostaining; 4-well plate with coverslip cell senescence: 4-well plate).

Mouse neonatal cardiomyocytes were characterized by immunostaining with α-actinin (Sigma, A7811) antibody.

**Conditioned medium collection and concentration**

Passage 4, 90% confluent cells were cultured in 10 cm plate. The growth medium was aspirated and the cells were washed three times with PBS, and then 8 mL DMEM (GIBCO, 21063-029) containing neither FBS nor PS was added. The cells were incubate under hypoxic condition (1% O_2_, 4% CO_2_) for 24 hours. The medium was collected, cleared by centrifugation, filtered by 0.22 μm millipore filter and then placed into an Amicon Ultra-4 Centrifugal Filter Unit (Millipore, UFC801024). The conditioned medium was purified and concentrated according to the manual with concentration factor of 10 times.

**Cell viability-MTT assay**

To test the differences between MSCe and MSC-ERBB4 in paracrine function, we used the concentrated conditioned medium to support cardiomyocyte cell growth under hypoxic condition (1% O_2_, 4% CO_2_) for 24 hours. In each well of a 96-well plate, we added 200 μL conditioned medium on 5 x 10^3^ cardiomyocytes. Cell viability was determined using a 96-well format with MTT assays (Sigma, M2003). To validate the role of self-secret NRG1 in MSC-ERBB4, anti-NRG1 antibody (Santa Cruz, SC-348) at concentration of 10 μg/mL was employed into the conditioned medium of MSC-ERBB4. MTT was added to each well. Plates were incubated for 4 hours in the absence of light before the medium was removed, and precipitates were re-suspended in 50 µL DMSO. Absorbance at 570 nm was measured using microplate reader. All experiments were carried out using three biological replicates.

**Cell proliferation**

Cardiomyocytes were cultured in conditioned medium of MSCe or MSC-ERBB4. To validate the role of self-secret NRG1 in MSC-ERBB4, anti-NRG1 antibody (Santa Cruz, SC-348) at concentration of 10 μg/mL was employed into the conditioned medium of MSC-ERBB4. Cardiomyocytes proliferation was determined by immunostaining against Ki67, a marker located in the neucleus indicating active phases of the cell cycle.

**Cell senescence-associated β-galactosidase staining**

This experiment was to determine if the secreta of MSCe and MSC-ERBB4 could protect cardiomyocytes from cell senescence under hypoxic challenge. Cardiomyocytes at passage 0 were seeded in 24-well plate at a density of 2 x 10^4^/per well with conditioned medium collected from MSCe or MSC-ERBB4 and kept under hypoxia for 24 hours. To validate the role of self-secret NRG1 in MSC-ERBB4, another group with anti-NRG1 antibody (Santa Cruz, SC-348) at concentration of 10 μg/mL in the conditioned medium of MSC-ERBB4 was employed. Then the senescent status was verified by *in situ* staining for senescence-associated β-galactosidase (SA-β-gal) (Millipore, KAA002) according to the manufacture’s instruction. Briefly, after washing step, the cells were fixed by 4% formaldehyde for 10 minutes. After another washing step with PBS, cells were incubated with SA-β-gal for 24 hours at 37° C without CO_2_, and protected from the light. The reaction was stopped by the addition of PBS. Senescent cells were shown in a distinctive blue color. For each group we have triplicate wells with 8 random sights each well.

**Western blotting**

Cells and tissue specimens were harvested and lysed in RIPA buffer (Sigma, R0278) supplemented with complete ULTRA tablets (ROCHE, 5892970001). The protein concentrations were measured by Bradford assay (BIO-RAD, 5000202). A total amount of 30 μg protein of each sample was loaded. Samples were separated by SDS/PAGE and then transferred to a PVDF membrane. Membranes were blocked in 5% wt/vol fat-free milk (BIO-RAD, 170-6404) before incubation with primary antibody overnight in 4° C. The relevant antibodies included NRG1 (Santa Cruz, SC-348), ERBB4 (Abcam, ab19391), p-ERBB4 (Abcam, ab109273), β-actin (Santa Cruz, SC-47778), Bcl-2 (cell-signaling, 2870), p-Akt (cell-signaling, 9611), t-Akt (cell-signaling, 9272) and GFP (Santa Cruz, SC-8334). Membranes were then blotted with appropriate HRP-conjugated secondary antibodies, including anti-rat (Santa Cruz, SC-2006), anti-goat (Santa Cruz, SC-2768), anti-mouse (Santa Cruz, SC-358920) and anti-rabbit (Santa Cruz, SC-2030) with a 1:1000 dilution. Bands were detected by enhanced chemiluminescence (GE Healthcare, RPN2232).

**Elisa assay**

Elisa was employed to evaluate NRG1 (USCN, E91866Mu) release in concentrated conditioned as per manuals. All groups were carried out using three biological replicates.

**Multiple transfections**

To validate the direct relationship between ERBB4 overexpression and NRG1 up-regulation, we designed a multiple transient transfection experiment. MSCs at 30~40% confluence were transfected with pER4-GFP for the 1^st^ time using lipofectamine 2000 at day 0. After 48 hours, a subset of cells were collected for flow cytometry and Western blotting against NRG1, and the rest were subjected to the 2^nd^ time of transfection. Repeat this process until the 5^th^ transfection was complete. The positive GFP population showed in flow cytometry indirectly reflected the proportion of cells with ERBB4 expression. With the same batch of cells, NRG1 expression was detected by Western blotting. The experiment was performed three times.

**Subcutaneous tumor model**

For tumor model, 1 x 10^6^ MSCe or MSC-ERBB4 were transplanted subcutaneously into NOD-SCID mice, with mouse embryonic cells inoculated to the other side of the animal as controls ( N = 4 ). Mice were kept under observation for 8 weeks.

**Statistical Analysis**

Values are expressed as mean ±standard error of mean (SEM). Student t-test or one or two-way ANOVA with the Bonferroni post-hoc test were done to determine statistical significance (*p*<0.05).

| **Supplementary Table I. List of primers** | | | |
| --- | --- | --- | --- |
|  | **Forward (5'to 3')** | **Reverse (5'to 3')** | **Size(bp)** |
| **LPL** | TAACTGCCACTTCAACCACA | AATCAGCGTCATCAGGAGA | 419 |
| **Acan** | CAAGAAATCGAATCCCCAAATC | ACTTAGTCCACCCCTCCTCACAT | 166 |
| **Pparg** | TTTCAAGGGTGCCAGTTTCG | GGTGGGACTTTCCTGCTAATACAA | 606 |
| **Col2a1** | CCGAGTGGAAGAGCGGAGAC | CAGTGGACAGTAGACGGAGGAAAG | 279 |
| **Bglap** | CCCAGACCTAGCAGACACCATG | TGTTCACTACCTTATTGCCCTCC | 155 |
| **Alpl** | TGGACGGTGAACGGGAAAA | AGCACAGCCAGTGGAAGCAG | 283 |
| **ERBB2** | GAGAACACCGAGGTCGCTTAG | CCCTTGCCATACGGGAGAA | 513 |
| **ERBB3** | GGACAGTGCCTACCATTCG | CGGAGTCAGTGGCTTCTAAAC | 581 |
| **ERBB4** | TTCCAACCCGAGAAATCCCC | TGTCTTCCAAATCCTCTTCATCCA | 276 |
| **NRG1** | CCTCCTTTTCCACCAgTCACTACA | ACAGAGGGCATGGACACCGT | 409 |
| **β-actin** | AGAGGGAAATCGTGCGTGAC | TGCTGGAAGGTGGACAGTGAG | 448 |

**Supplementary Figure Legends**

**Supplementary Figure I. Characterization of MSCs.**

**A**, The spindle-shaped morphology of MSCs in culture (**Ai**) and their multiple differentiation capability (**Aii, Aiii, and Aiv**). Scale bar = 200 µm. **B,** The transcription levels of lineage specific genes were measured using RT- PCR. β-actin was used as a loading control. **C,** Immunophenotypic profile of MSCs. Representative flow cytometry histograms in the sixth passage showed the positive expression of surface molecules SCA1, CD90.2, and CD44 and negative expression of hematopoietic cell lineage-specific antigens CD34, CD45, and CD117. The control cells were labeled without using primary antibodies (unshaded). The experiment was repeated 3 times and the statistics are shown.

**Supplementary Figure II. Plasmid map of pGFP and pER4-GFP provided by GeneCopoeia.**

**Supplementary Figure III. Characterization of mouse cardiomyocytes**

Isolated mouse cardiomyocytes were positive for mature cardiomyocytes marker α-actinin.

**Supplementary References**

1. al Yacoub N, Romanowska M, Haritonova N, Foerster J. Optimized production and concentration of lentiviral vectors containing large inserts. *The journal of gene medicine*. 2007;9:579-584

2. Pacher P, Nagayama T, Mukhopadhyay P, Batkai S, Kass DA. Measurement of cardiac function using pressure-volume conductance catheter technique in mice and rats. *Nat Protoc*. 2008;3:1422-1434
